# Macrophage-Derived Small Extracellular Vesicles in Multiple Diseases: Biogenesis, Function, and Therapeutic Applications

**DOI:** 10.3389/fcell.2022.913110

**Published:** 2022-06-27

**Authors:** Jingyao Ye, Xuehong Liu

**Affiliations:** ^1^ Shandong University of Traditional Chinese Medicine, Jinan, China; ^2^ The Third School of Clinical Medicine of Guangzhou University of Chinese Medicine, Guangzhou, China

**Keywords:** macrophagederived small extracellular vesicles, polarization, multiple diseases, biological functions, applications

## Abstract

Macrophages (*Mφs*), as immune cells, play a pivotal role against pathogens and many diseases, such as cancer, inflammation, cardiovascular diseases, orthopedic diseases, and metabolic disorders. In recent years, an increasing number of studies have shown that small extracellular vesicles (sEVs) derived from *Mφs* (M-sEVs) play important roles in these diseases, suggesting that *Mφs* carry out their physiological functions through sEVs. This paper reviews the mechanisms underlying M-sEVs production *via* different forms of polarization and their biological functions in multiple diseases. In addition, the prospects of M-sEVs in disease diagnosis and treatment are described.

## Introduction

Extracellular vesicles (EVs) are small vesicles released by all cells including prokaryotes and eukaryotes. EVs are categorized into exosomes (Exos), microvesicles (MV), apoptotic bodies, exomeres and large oncosomes based on their different origins and sizes. The most common method to distinguish EV subtypes is their size. In the literature related to macrophage-derived EVs, Exos are mostly involved. However, due to methodological difficulties of separation, it is worth noting that the term “exosomes”, even if widely used, has been suggested to be replaced by the term “small EVs (sEVs)” (Théry et al., 2018). Therefore, we primarily focus on the biological roles of macrophage-derived sEVs in this review. As one of the extracellular vesicles, recent studies have shown that sEVs contain many cellular components, including RNA, lipids, metabolites, cytoplasm, and cell surface proteins. DNA is a controversial molecule in sEVs, and previous studies revealed that all cellular contents may all exist in sEVs, while recent studies have shown that double-stranded DNA (dsDNA) and DNA-binding histones are not carried by sEVs (Jeppesen et al., 2019), which indicates that more studies on sEVs are needed.

sEVs are widely distributed in a variety of body fluids, including blood, urine, peritoneal fluid, synovial fluid, and breast milk. The physiological purpose of producing sEVs is largely different, which was initially described as a means of eliminating unneeded compounds from the cell, while recently, they are proven to act as signaling vehicles in pathological developments. sEVs have great heterogeneity. This may be due to their different sizes, numbers, content, and organs and tissues of origin, such as cancer cells or stimulation by external stimuli like radiotherapy, which leads to different biological functions. The combination of all these features likely leads to a higher level of sEV complexity and heterogeneity, as well as orientation to specific organs and uptake by specific cell types. They can affect different biological processes, including immune response, cell proliferation, cell migration, and angiogenesis, as well as promote or inhibit a variety of diseases, such as orthopedic disorders, viral infections, cardiovascular disease, metabolic disorders and cancer progression (McKelvey et al., 2015; Mathieu et al., 2019; Kalluri and LeBleu, 2020). sEVs are considered potential new therapeutic tools for drug delivery systems or biomarkers due to their special lipid bilayer.

**FIGURE 1:**
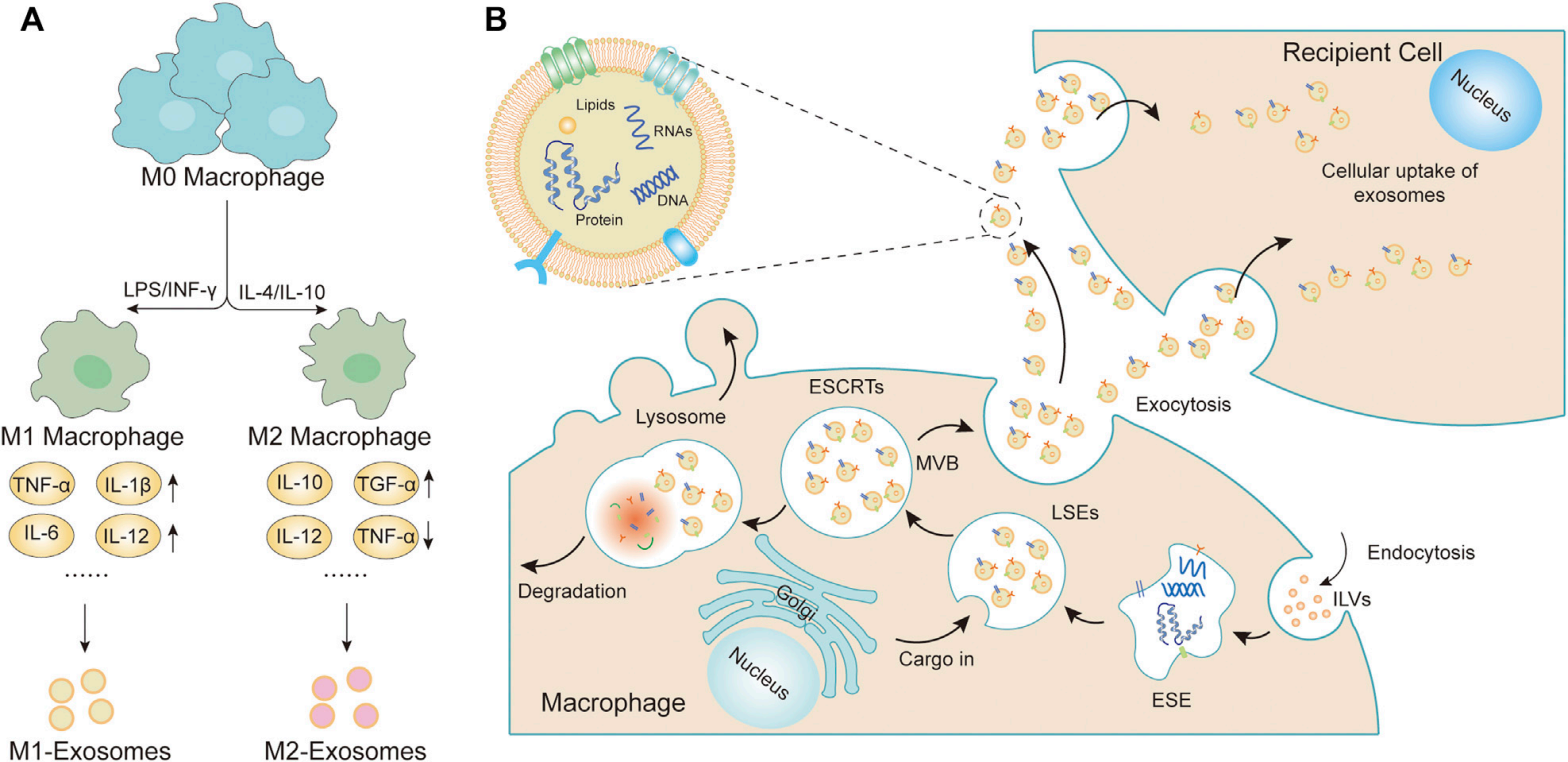
Macrophage polarization and formation of sEVs. **(A)** *Mφs* could be roughly divided into two subtypes (M1 *Mφs* and M2 *Mφs*) depending on the different microenvironmental stimuli. M1 *Mφs* are typically induced by IFN-γ or LPS while M2 *Mφs* are induced by IL-4 or IL-10. M1 *Mφs*-sEVs secrete high levels of proinflammatory cytokines, such as TNF-α, IL-1β, IL-6, IL-12, and IL-23, promoting the inflammatory and cytotoxic responses. M2 *Mφs*-sEVs can not only directly inhibit pro-inflammatory enzymes and cytokines, such as IL-12 and TNF-α, to achieve anti-inflammatory effects but can also display higher levels of certain anti-inflammatory factors, such as IL-10 and TGF-β, thereby resolving deleterious inflammatory conditions. **(B)** The cytoplasmic membrane of the *Mφs* initially invaginates to form endocytic vesicles, and multiple endocytic vesicles fuse to form early-sorting endosomes (ESEs). The ESEs then invaginate, encapsulating intracellular material in the process, further transforming into late-sorting endosomes (LSEs), which are known as multivesicular bodies (MVBs). MVBs then fuse with the cytoplasmic membrane and releases EVs into the extracellular space.

As innate immune cells, macrophages (*Mφs*) have high heterogeneity and plasticity, which not only play a clear role in the main response to pathogens, but also play a significant role in tissue homeostasis, coordination of adaptive immune response, inflammation, resolution, and repair. Their phenotype and function are regulated by the surrounding microenvironment. *Mφs* can be divided into classically activated *Mφs* (M1 *Mφs*) and alternately activated *Mφs* (M2 *Mφs*) according to the expressed cell surface markers, production of specific factors, and biological activity (Martinez et al., 2009). M1 *Mφs* have powerful antibacterial and tumor-inhibiting functions and are believed to play an important role in the host defense mechanisms against pathogens. They are activated by infection-related metabolites, such as lipopolysaccharide (LPS) and interferon-γ (INF-γ), inducing M1 *Mφs* to secrete high levels of pro-inflammatory cytokines, such as tumor necrosis factor-α (TNF-α), cyclooxygenase-21, interleukin-6 (IL-6), and IL-12, which induce the inflammatory response. Unlike M1 *Mφs*, M2 *Mφs* regulate inflammation after tissue damage and can be further divided into four different types depending on the type of stimulation: M2a, M2b, M2c, and M2d *Mφs*. Polarized by cytokines IL-4/IL-10, M2 *Mφs* are induced to secrete anti-inflammatory factors, thereby reducing the inflammatory response, with an anti-inflammatory and strong phagocytosis effect, eliminating apoptotic cells, which can be used to treat chronic infections and wounds (Chinetti-Gbaguidi et al., 2015; Murray and Julius, 2017; Atri et al., 2018; Funes et al., 2018) (see Figure 1). The timing, quantity, and degree of M1/M2 *Mφs* polarization control the fate of various diseases.

Similar to other EVs, sEVs derived from *Mφs* (M-sEVs) also carry biological information and play an important regulatory role in a variety of diseases, and have biological compatibility, so they can be used as carriers of drug delivery. In this review, M-sEVs are discussed in different diseases in the mechanism of formation, classification, and function. In addition, the application of M-sEVs as a drug, gene, and protein delivery tool in bioengineering is described, and the potential therapeutic targets of M-sEVs for various diseases are further discussed.

## Formation Mechanisms of M-sEVs

The formation of M-sEVs is similar to that of most cell-derived sEVs, which is precisely regulated and involves multiple proteins. Intraluminal vesicles (ILVs) are present on the plasma membrane of *Mφs* (Kalluri and LeBleu, 2020). Multiple ILVs fuse with early-sorting endosomes (ESEs) and often the newly formed ESEs directly merge with the existing ones. The trans-Golgi network and endoplasmic reticulum also contribute to the formation and content of ESEs. Later ESEs invaginate, encapsulating intracellular material and maturing into late-sorting endosomes. They are further processed and mature to form multivesicular bodies (MVBs). MVBs can either fuse with lysosomes or autophagosomes for degradation or fuse with the plasma membrane to release ILVs as sEVs. A variety of complex proteins are involved in the origin and biogenesis of sEVs (Kalluri, 2016; Hessvik and Llorente, 2018; van Niel et al., 2018; Willms et al., 2018; Mathieu et al., 2019; McAndrews and Kalluri, 2019) (see Figure 1). The ESCRT pathway is the most studied formation mechanism of ILVs and MVBs. It not only controls the dissociation of sEVs and participates in the packaging of biomolecules into sEVs but also mediates proteins in MVBs through an independent pathway. However, the precise roles and functions of these proteins in sEV biogenesis warrant further research. Usually, most mature MVBs are decomposed by lysosomes, while undecomposed MVBs are released into the extracellular environment in the form of sEVs with the help of Rab protein and small GTPases. Therefore, when the lysosomal-associated membrane protein (LAMp) 1/2 is downregulated, more M-sEVs production can be observed due to lysosome dysfunction (Liu et al., 2020a; Shan et al., 2021). Because M-sEVs are very sensitive to the extracellular and intracellular environment, different sEVs are formed under different endogenous and exogenous stimuli, such as radiation, aging, hypoxia, or the presence of metal ions or cellular stress. Three types of M-sEVs are currently recognized, including unpolarized M0 *Mφs*-derived sEVs (M0-sEVs) and polarized M1 and M2 *Mφs*-derived sEVs (M1-sEVs and M2-sEVs) (Funes et al., 2018). Known sEV isolation methods could be applied to the isolation of sEVs from *Mφs.* Currently, sEVs isolation methods have been established, such as ultracentrifugation techniques, polymer precipitation, and size-based isolation technique and each method has advantages and disadvantages. ultracentrifugation is the most common isolation method it needs major improvements due to its time-consuming nature, high cost, and other disadvantages; therefore, more studies are needed to improve the separation efficiency and enrichment. So far, there are many studies on microRNAs (miRNAs), long noncoding RNAs (lncRNAs), and proteins in M-sEVs (see Tables 1‐3). It is confirmed that miRNAs regulate the biological effects of cancer cells by directly binding to the 3′UTR of mRNAs.However, few studies are available on other components, such as mRNAs, tRNAs, and ribosomes. M-sEVs with different phenotypes contain different biological information and thus perform different functions. These findings have important implications for understanding the role of different cell types and environments in regulating sEV release.

**TABLE 1:** The same miRNA/lncRNA exerts different biological functions.

MiRNA	Donor cells	Recipient cells	Biological function	Targets	Reference
MiRNA-223	*Mφs*	HCC, Cancer cells	Inhibit proliferation of cells	STMN1 and IGF-1R	Aucher et al. (2013)
	*Mφs*	GC, Cancer cells	Promote metastasis of cells, alter actin cytoskeleton, upregulate multiple proteins associated with EMT	PTEN/PI3K/AKT	Zheng et al. (2020)
	*Mφs*	GC, Cancer cells	Promote doxorubicin resistance	F-box/FBXW7	Gao et al. (2020)
	Hypoxic *Mφs*	EOC, Cancer cells	Promote chemotherapy resistance	PTEN/PI3K/AKT	Zhu et al. (2019a)
	IL-4 activated M2 *Mφs*	BCC, Cancer cells	Promote cell invasion	Mef2c/β-catenin	Yang et al. (2011)
MiRNA-155	M2 TAMs	NSCLC, Cancer cells	Promote cell migration, invasion, and EMT	RASSF4	Li et al. (2021b)
	M1 *Mφs*	MI, cardiac fibroblast	Suppress fibroblast proliferation and promote fibroblast inflammation	Sos1	Wang et al. (2017)
	M1 *Mφs*	MI, endothelial cells	Reduce the angiogenic ability of ECs, aggravate myocardial injury, and restrain cardiac healing	RAC1/PAK_1/2_ and SirT1/AMPK_2α-e_NOS	Liu et al. (2020d)
	*Mφs*	UCM, cardiomyocytes	Promote cardiomyocyte pyroptosis, cardiac hypertrophy, and fibrosis	FoxO3a	Wang et al. (2020b)
	Obese Adipose Tissue *Mφs*	T2DM, adipocytes and muscle cells	Glucose intolerance and insulin resistance	*PPARG*	Liu et al. (2019)
MiRNA-155-5p	M2 TAMs	PDAC, endothelial cells	Promote the growth and angiogenesis of tumors	E2F2	Yang et al. (2021b)
	M2 TAMs	CC, Cancer cells	Promote cell proliferation and anti-apoptosis	ZC3H12B-mediated IL-6	Ma et al. (2021)
	TAMs	IAs, SMCs	Promote proliferation and migration of SMCs	Gremlin 1	Feng et al. (2019)
MiRNA-27a-3p	M2 *Mφs*	HCC, Cancer cells	Promote tumorigenicity	TXNIP	Li et al. (2021a)
	M2 TAMs	IH, Hemangioma stem cells	Inhibit sensitivity to propranolol	DKK2	Liu et al. (2021a)
MiRNA-21-5p (MiRNA-21a-5p)	M2 *Mφs*	PC, Cancer cells	Promote the differentiation and activity of PC stem cells	KLF3	Chang et al. (2021)
	M1 *Mφs*	MI, cardiomyocyte	Promote myocardial fibrosis and ventricular remodeling	TIMP3	Dong et al. (2021)
	LPS activated M1 *Mφs*	Asthma, trachea epithelial cells	Promoting EMT and airway remodeling	TGFβ1/Smad7	Li et al. (2021c)
	Bone marrow *Mφs*	TI, tendon cells	Induce fibrogenesis in tenocytes	Smad7/TGF-β1	Cui et al. (2019)
	TAMs	EOC, T cell	Result in a Treg/Th17 imbalance and promot tumor progression	STAT3	Zhou et al. (2018)
	M1 *Mφs*	UC, enterocytes	Induce destruction of the intestinal mucosal epithelium	E-cadherin	Lu et al. (2021)
	M1 *Mφs*	Bone fracture, BMSCs	Romote osteogenesis of BMSCs	Not mention	Liu et al. (2022)

HCC, hepatocellular carcinoma; GC, gastric carcinoma; EMT, epithelial-mesenchymal transition; BCC, breast carcinoma; EOC, epithelial ovarian cancer; MI, myocardial infarction; NSCLC, non-small-cell lung cancer; ECs, endothelial cells; UCM, uremic cardiomyopathy; T2DM, type 2 diabetes mellitus; CC, colon cancer; WDR82, WD, repeat domain 82; MB, medulloblastoma; PDAC, pancreatic ductal adenocarcinoma; IA, intracranial aneurysm; SMCs, smooth muscle cells; UIAs, unruptured intracranial aneurysms; UC, ulcerative colitis; IH, infantile hemangioma; PC, Pancreatic cancer; EC, esophageal cancer; TI, tendon injury; BMSCs, bone mesenchymal stem cells; DN, Diabetic nephropathy.

**TABLE 2:** Other contents (including lncRNAs, circRNAs, proteins, mRNAs) exert different biological functions.

Content	Donor cells	Recipient cells	Biological function	Targets	Reference
lncRNA AGAP2-AS1	M2 *Mφs*	NSCLC, Cancer cells	Strengthen radioresistance and promote the malignant behavior of cells	miR-296/NOTCH2	Zhang et al. (2021a)
ApoE	M2 TAMs	GC, Cancer cells	Reshape the migration of cytoskeleton support	PI3K/Akt	Zheng et al. (2018)
hsa_circ_0001610	M2 TAMs	EC, Cancer cells	Reduce the radiosensitivity of endometrial cancer cells	miR-139-5p/cyclin B1	Gu et al. (2021)
lncRNA CHL1-AS1	peritoneal *Mφs*	EM, ectopic endometrial stromal cells	Enhance the proliferation, migration, and invasion of ectopic endometrial stromal cells	miR-610/MDM2	Liu et al. (2021b)
ADAM15	LPS activated M1 *Mφs*	EOC, Cancer cells	Suppress tumor progression without triggering other immune cells	Not mention	Lee et al. (2012)
SPP1	silica-exposed *Mφs*	Silicosis, fibroblasts	Activate the downstream cascade and lead to myofibroblast transition	Not mention	Huang et al. (2021b)
AT1R	Ang II-stimulated *Mφs*	Silicosis, fibroblasts	Promote collagen synthesis, fibroblast activation, and pulmonary fibrosis	TGF-β/SMad2/3	Sun et al. (2021)
circRNA-Ep400	M2 *Mφs*	TI, tendon cells	Promote fibrosis, proliferation, and migration of fibroblasts and tendinocytes	miR-15b-5p/FGF1/7/9	Da-Wa et al. (2021)
LncRNA SBF2-AS1	M2 *Mφs*	PC, Cancer cells	Promoting the tumorigenic ability of PC cells	miRNA-122-5p/XIAP	Yin et al. (2020)
LncRNA AFAP1-AS1	M2 *Mφs*	EC, Cancer cells	Promote the migration, invasion, and lung metastasis	miRNA-26a/ATF2	Lee et al. (2012)
TGF-β1 mRNA	high glucose-induced *Mφs*	DN, mesangial cell	Induce mesangial cell proliferation, promote fibrosis, and accumulation of inflammatory factors	TGF-β1/Smad3	Zhu et al. (2019c)
LFA-1	*Mφs*	inflamed brain, ECs	Improve drug delivery to the brain for future treatments	ICAM-1	Ying et al. (2020)

**TABLE 3:** Other miRNA exerts different biological functions.

MiRNA	Donor cells	Recipient cells	Biological function	Targets	Reference
MiRNA-326	M1 *Mφs*	HCC, Cancer cells	Reduce cancer cell proliferation, colony formation, migration, and invasion	NF-κB	Bai et al. (2020)
miRNA-142	*Mφs*	HCC, Cancer cells	Inhibit cancer cell proliferation	Not mention	Li et al. (2021a)
miRNA-660-5p	M2 *Mφs*	HCC, Cancer cells	Promote cancer stemness, drug resistance, migration, invasion, and tumorigenicity	KLF3	Tian et al. (2021)
MiRNA-92a-2-5p	M2 TAMs	HCC, Cancer cells	Increase the invasion of HCC cells	AR/PHLPP/P-Akt/β-catenin	Liu et al. (2020b)
MiRNA-125a/b	TAMs	HCC, Cancer cells	Promote HCC cell proliferation and stem cell properties	CD90	Wang et al. (2019a)
MiRNA-181a-5p	M1 TAMs	LUAD, Cancer cells	Promote cancer cells apoptosis	ETS1/STK16	Wang et al. (2022a)
MiRNA-942	M2 TAMs	LUAD, Cancer cells	Promote angiogenesis, and promote the progression of LUAD	FOXO1/β-catenin	Wei et al. (2022)
miRNA-155 miRNA-196a-5p	M2 TAMs	NSCLC, Cancer cells	Promote migration, invasion of cancer cells	RASSF4	Li et al. (2021b)
miRNA-501-3p	M2 TAMs	LC, Cancer cells	Promote the progression of diseases	WDR82	Song et al. (2022)
miRNA-155-3p	M2 *Mφs*	MB, Cancer cells			Lei et al. (2021)
miRNA-3679-5R	M2a TAMs	LC, Cancer cells	Promote chemoresistance in lung cancer	NEDD4L/C-Myc	Wang et al. (2020a)
miRNA-16-5p	M1 *Mφs*	GC, Cancer cells	Activate T cell immune response and inhibite the proliferation of GC cells	PD-L1	Li et al. (2020b)
miRNA-21	M2 *Mφs*	GC, Cancer cells	Promote cisplatin resistance and suppresses cell apoptosis	PTEN/PI3K/AKT	Zheng et al. (2017)
miRNA-487a	M2 *Mφs*	GC, Cancer cells	Induce the proliferation and tumorigenesis of GC cells	TIA1	Yang et al. (2021a)
miRNA-588	M2 *Mφs*	GC, Cancer cells	Lead to cisplatin resistance in GC cells	CYLD	Cui et al. (2021)
miRNA-221-3p	Peritoneal M2 TAMs	EOC, Cancer cells	Promote the progression of EOC	CDKN1B	Li and Tang, (2020)
miRNA-29a-3p	TAMs	EOC, T cell	Result in a Treg/Th17 imbalance and promot tumor progression	STAT3	Zhou et al. (2018)
miRNA-7	TWEAK-stimulated *Mφs*	EOC, Cancer cells	Inhibit the metastasis of EOC cells	EGFR/AKT/ERK1/2	Hu et al. (2017)
miR-146b-5p	TAMs in ascites	EOC, endothelial cell	Inhibit endothelial cell migration	RAF6/NF-κβ/MMP-2	Wu et al. (2017)
miRNA-503-3p	*Mφs*	BCC, Cancer cells	Promote the malignant phenotype of BCC cells and promote tumor progression	DACT2, Wnt/β-catenin,glycolysis,OXPHOS	Huang et al. (2021a)
miRNA-192-5p	M2 TAMs	EC, Cancer cells	Inhibit apoptosis and promoting tumor development	IRAK1/NF-κβ	Wang et al. (2022b)
miRNA-22-3p	Peritoneal *Mφs*	EM, ectopic endometrial stromal cells	Enhance the proliferation, migration, and invasion of ectopic endometrial stromal cells	SIRT1/NF-κB	Zhang et al. (2020)
miRNA-501-3p	M2 TAMs	PC, Cancer cells	Promote the differentiation and activity of PC stem cells	TGF-β/TGFBR3	Yin et al. (2019)
miRNA-365	M2 TAMs	PDAC, Cancer cells	Promote gemcitabine resistance in PDAC	NTP/CDA	Binenbaum et al. (2018)
miRNA-17-3p	AngII-stimulated *Mφs*	Hpertension		ICAM1/PAI-1	Osada-Oka et al. (2017)
miRNA-221	M2 TAMs	PDAC, endothelial cells	Promote the growth and angiogenesis of tumors	E2F2	Yang et al. (2021b)
miRNA-31-5p	M2 *Mφs*	OSCC, Cancer cells	Support OSCC growth	LATS2 and Hippo	Yuan et al. (2021)
miRNA-183-5p	M2 *Mφs*	CC, Cancer cells	Promote cancer cell proliferation, invasion, and metastasis	THEM4-mediated PI3K/AKT and NF-κβ	Zhang et al. (2021b)
miRNA-99a	IL-4 activated Bone marrow M2 *Mφs*	AS, HSCs, myeloid cells, multipotent progenitors	Reduce hematopoiesis and the inflammatory state	TNF-α/NF-κB	Bouchareychas et al. (2020)
miRNA-146b					
miRNA-378a-3p					
miRNA-106a-3p	Oxidized LDL induce *Mφs*	AS, VSMCs	Promote cell proliferation and repress cell apoptosis	CASP9/caspase	Liu et al. (2020c)
miRNA-222	H/SD induce M1 *Mφs*	MI, BMSC	Reduce BMSC viability and migration, increased BMSC apoptosis	B-cell lymphoma	Qi et al. (2021)
miRNA-29a	H/Rn-induced and LPS-induced M1 *Mφs*	Ischemia-reperfusion injury, CM	Promote CM pyroapoptosis	MCL-1	Wang et al. (2021b)
MiRNA-148a	M2 *Mφs*	MI, CM	Reduce myocardial ischemia-reperfusion injury	TXNIP and TLR4/NF-κβ/NLRP3	Dai et al. (2020)
miRNA-1271-5p	M2 *Mφs*	AMI, cardiomyocytes	Reduce cardiac apoptosis and promotes cardiac repair	Sox6	Long et al. (2021)
miRNA-328	M2 *Mφs*	PF, lung fibroblasts	Promote the progression of pulmonary fibrosis	FAM13A	Yao et al. (2019)
miRNA-142-3p	*Mφs*	PF, alveolar epithelial cells and lung fibroblasts	Fight against pulmonary fibrosis progression	TGFβ-R1	Guiot et al. (2020)
miRNA-370	M2 *Mφs*	Asthma, ASMCs	Reduce lung injury and inflammatory, inhibit ASMC proliferation and airway remodeling	FGF1and MAPK/STAT1	Li et al. (2021d)
miRNA-153-3p	Decidual *Mφs*s	URSA, trophoblastic cells	Inhibit the proliferation and migration of trophoblastic cells and regulate the behavior of trophoblastic cells	IDO/STAT3	Ying et al. (2020)
miRNA-5106	M2 *Mφs*	Bone fracture, BMSCs	Promote osteoblast differentiation and bone mineral deposition, and accelerate fracture healing	SIK2 and SIK3	Xiong et al. (2020)
miRNA-501	M2 *Mφs*	Bone fracture, myoblasts	Promote myotube formation and improves the inflammatory cell infiltration	YY1	Zhou et al. (2021)
miRNA-98	M1 *Mφs*	OP,osteoblast	Aggravate bone loss	DUSP1/JNK	Yu et al. (2021)
miRNA-210	High Glucose-Induced *Mφs*	T2DM, adipocyte	Promotes insulin resistance and obesity, and promotes diabetic obesity pathogenesis	NDUFA4	Tian et al. (2020)
miRNA-29a	Obese adipose tissue *Mφs*	T2DM, adipocyte	Impel glucose uptake, promote glucose output, reduce cellular and systemic insulin sensitivity, and promote obesity-induced insulin resistance	PPAR-δ	Liu et al. (2019)
MiRNA-143-5p	High-fat diet induced BMM1 *Mφs*	T2DM, liver cells	Promote insulin resistance	Mkp5	Li et al. (2021e)
miRNA-212-5p	High-fat diet induced BMM1 *Mφs*	T2DM, islet β cells	Disrupt insulin secretion and glucose intolerance	Akt/GSK-3β/β-catenin	Qian et al. (2021)
miRNA-690	BMM1 *Mφs*	T2DM, adipocytes, myocytes, hepatocytes	Improve insulin sensitivity, reduce obesity-related adipose tissue inflammation and promotes repolarization of Mφs from the M1 to M2 phenotype	NADK	Ying et al. (2021)
miRNA-144-5p	dBMDM *Mφs*	T2DM with fracture, BMSCs	Prevent fracture healing	Smad1	Zhang et al. (2021c)
MiRNA-25-3p	High Glucose-Induced M2 *Mφs*	DN, GVEs	Activate the autophagy of GVEs, protect the GVEs from high glucose-induced damage	DUSP1	Huang et al. (2020)

OSCC, oral squamous cell carcinoma; HSC, hematopoietic stem cell; AS, atherosclerosis; VSMCs, human vascular smooth muscle cells; H/SD, hypoxia/serum deprivation PF, Pulmonary fibrosis ASMCs, airway smooth muscle cells; URSA, unexplained recurrent spontaneous abortion OP, osteoporosis; GVEs, glomerular visceral epithelial cells; BMM, bone marrow-derived macrophages; dBMDM, diabetic bone marrow-derived macrophages; CM, cardiomyocyte; MB, medulloblastoma; LUAD, lung adenocarcinoma; EM, endometriosis.

## Role of M-sEVs in Cancer

The tumor microenvironment (TME) plays an important role in the development of various types of tumors. It is home to many immune cells. In this microenvironment, *Mφs*, also known as tumor-associated macrophages (TAMs), are the most abundant population of immune cells. Recent studies have revealed that TAMs not only play an important role in tumor formation and development but are also closely related to drug resistance, prognosis, and survival rate. Different types of polarized *Mφs* secrete various sEVs, and different components of sEVs affect disease development in various forms under different pathological conditions. M1 *Mφs* have an anti-tumor function, while M2 *Mφs* can promote tumor metastasis. In most malignant tumors, the physiological function of M2 *Mφs* is more prominent than that of M1 *Mφs*, which explains the poor prognosis of malignant tumors. However, the underlying mechanisms of how two different types of M*-*sEVs exert their effects is not entirely known. Here, we review how different sEVs derived from TAMs (TAMs-sEVs) play roles in cancers.

### Liver-Related Cancers

Liver cancers include hepatocellular carcinoma (HCC), intrahepatic cholangiocarcinoma, and other rare types. This article focuses on HCC with a high incidence. MiRNA-326, which can suppress the expression of tumor-related genes, is downregulated in HCC cells, but found in high levels in M1-sEVs, thereby reducing cell proliferation, colony formation, migration, and invasion, as well as NF-κB expression, promoting apoptosis of HCC cells, reducing the volume and weight of HCC tumors (Bai et al., 2020). However, in this study, THP-1 cells were induced into M1 *Mφs in vitro*, and the specific polarization of *Mφs in vivo* needs more research. Both miRNA-142 and miRNA-223 are endogenously expressed in *Mφs* but not in HCC cells, and these two miRNAs affect the post-transcriptional regulation of proteins in HCC after effective transfer through sEVs upon cell-cell contact. Reduced expression of both reporter proteins and endogenous expressions of stathmin-1 (STMN1) and insulin-like growth factor-1 receptor (IGF-1R) functionally inhibit the proliferation of these cancer cells. However, miRNAs are transferred *via* direct contact between *Mφs* and HCCs, and sEVs are not a major pathway for intercellular transfer of miRNA. This indicates the limitations of sEVs in certain diseases (Aucher et al., 2013). M-sEVs have an inhibitory effect on tumor development, but not always. For example, tumor-promoting miRNA-27a-3p and miRNA-660-5p loaded in M2-sEVs lead to the overexpression of these in HCC cells, respectively, downregulating thioredoxin-interacting protein (TXNIP) and Krüppel-like factor 3 (KLF3) that can inhibit tumor development. These results suggest that M2-sEVs promote cancer stemness, drug resistance, migration, invasion, and tumorigenicity in HCC *via* miRNA-27a-3p/TXNIP and miRNA-660-5p/KLF3 pathways; however, they do not affect the proliferation of cancer cells (Li et al., 2021a; Tian et al., 2021). In addition, M2-sEVs can contain high levels of miRNA-27a-3p and deliver it to hemangioma stem cells to inhibit their sensitivity to propranolol in infantile hemangioma (IH) by downregulating dickkopf-related protein 2 (DKK2) (Liu et al., 2021a). This suggests that the same factor has completely different physiological functions *via* different receptors, possibly related to the TME (see Table 1). MiRNA-92a-2-5p from M-sEVs can target the androgen receptor (AR) mRNA, inhibit AR translation, change AR/PHLPP/P-Akt/β-catenin signaling, and increase the invasion of HCC cells (Liu et al., 2020b). Since AR promotes the development of HCC in the early stages and inhibits it in the later stages, whether M-sEVs act on AR by secreting other miRNAs in the later stages and play different roles, warrants more research. Intriguingly, a low level of miRNA‐125a and miRNA-125b in M2-sEVs inhibit proliferation and stem cell characteristics of HCC cells by targeting CD90, a stem cell marker of HCC (Wang et al., 2019a). MiRNA-125a/b are different from the overexpressed miRNAs previously mentioned, suggesting that sometimes M-sEV miRNAs carrying out the biological functions are not dose-dependent. Together, these findings suggest that M-sEVs in the tumor microenvironment of HCC positively and negatively regulate the progression of HCC. Most studies on HCC show that M2 *Mφs* are higher in numbers than M1 *Mφs*, which may also be an important reason for the poor prognosis of HCC. Therefore, it is also a research direction whether the *in vitro* intervention of *Mφs* polarization into M1 may be applicable *in vivo* to inhibit the development of cancer, which also offers new ideas for the treatment of HCC.

### Lung-Related Cancers

As one of the malignant tumors, lung cancer (LC) has high morbidity and mortality worldwide (Siegel et al., 2020). Due to the lack of early detection methods, many patients are often diagnosed after metastases, greatly reducing survival (Strano et al., 2013). The E-twenty-six proto‐oncogene 1 transcription factor (ETS1) is involved in cell development, differentiation, and proliferation by directly regulating the expression of cytokine and chemokine-related genes (Dittmer, 2003; Garrett-Sinha, 2013; Taveirne et al., 2020). Serine/threonine kinase 16 (STK16), a membrane-associated kinase, is involved in the regulation of cell proliferation, apoptosis, sEV signaling pathways, and metabolism (Wang et al., 2019b). MiRNA-181a-5p delivered by M1-sEVs inhibit the expression of STK16 by targeting ETS1 to regulate cell viability and apoptosis in lung adenocarcinoma (LUAD), while *STK16* silencing significantly reduces the mass of nodules in tumor models, thus promoting the apoptosis of LUAD cells (Wang et al., 2022a). Serum miRNA-181a-5p was considered a potential noninvasive biomarker for the diagnosis and prognosis of patients with non-small cell lung cancer (NSCLC), while a further argument may be needed.

MiRNA-942 in M2-sEVs regulates Forkhead box transcription factor O1 (FOXO1) expression and further reduces the inhibition of β-catenin in LUAD cells, promotes angiogenesis, and promotes the progression of LUAD (Wei et al., 2022). It is well known that angiogenesis is important in tumor metastasis, thereby miRNA-942 provides a new therapeutic target for metastatic LUAD. Member 4 of the RAS-related domain family (RASSF4), which is abnormally expressed in human cancers, participates in carcinogenesis and has an inhibitory effect on biological processes (Han et al., 2016). RASSF4 effectively inhibits the proliferation and invasion of cancer cells, acting as an important tumor suppressor. However, miRNA-155 and miRNA-196a-5p of M2-sEVs negatively regulate the expression of RASSF4 in NSCLC and promote metastasis of NSCLC (Li et al., 2021b). In other studies, WD repeat domain 82 (WDR82) overexpression inhibited the metastasis of tumor cells, while miRNA-501-3p and miRNA-155-3p of M2-sEVs promoted the progression of LC and medulloblastoma (MB) by downregulating WDR82 (Lei et al., 2021; Song et al., 2022). This indicates that different miRNAs can act on the same target gene to produce similar physiological effects in the same or different diseases. M-sEVs cannot only directly affect tumor metastasis, proliferation, apoptosis, and other biological processes but also indirectly affect these by enhancing or inhibiting drug resistance. For example, Neuronally expressed developmentally downregulated 4 (NEDD4L), negatively regulated by miRNA-3679-5p of M2a-sEVs, is an E3 ligase that regulates ubiquitination and c-Myc degradation. NEDD4L plays its biological function mainly *via* mediating ubiquitination and degradation of target proteins in the endoplasmic reticulum, lysosomes, or proteasomes. Reduced expression of NEDD4L leads to reduced degradation of c-Myc. Stable c-Myc can promote aerobic glycolysis and induce drug resistance of LC cells. That is, M2-sEVs activate the miRNA-3679-5R/NEDD4L/C-Myc signaling pathway and induce drug resistance in LC (Wang et al., 2020a). Interestingly, sEVs have a unique structure that is biocompatible and can help drugs pass through natural barriers, demonstrating the importance of their content in disease development. Studies have proved that cisplatin delivery *via* M1-sEVs can enhance its anti-cancer effect in LC (Li et al., 2020a). Studies have also shown that M-sEVs cannot only affect disease progression through miRNAs but also affect the immune response of patients after chemotherapy through lncRNAs, such as the lncRNA AGAP2 antisense RNA 1 (AGAP2-AS1) located at 12q14.1, 1567 nucleotides in length. Its overexpression is associated with a poor prognosis of NSCLC and is a diagnostic biomarker for NSCLC (Luo et al., 2019; Tao et al., 2020) (see Table 2). M2-sEVs strengthen radioresistance of LC cells, promote the malignant behavior of LC cells, and reduce survival of patients with LC after radiotherapy inducing the overexpression of AGAP2-AS1, downregulating miR-296, and upregulating notch homolog protein 2 (NOTCH2) (Zhang et al., 2021a). This shows that the effect of M-sEVs on disease is not dependent on a single type of load but can be coordinated by sEVs simultaneously.

### Gastric-Related Cancers

Gastric carcinoma (GC) is a malignant tumor characterized by the growth of tumor cells in the stomach. In a previous pembrolizumab trial targeting programmed death ligand-1 (PD-L1), tumors shrank in more than 50% of patients with advanced GC, suggesting that PD-L1 is a promising target associated with GC immunotherapy (Matsueda and Graham, 2014). Studies have shown that miRNA-16-5p of M1-sEVs inhibits the formation of GC by reducing the expression of PD-L1, activating T cell immune response and inhibiting the proliferation of GC cells (Li et al., 2020b). However, M2-sEVs may upregulate PD-L1 expression *via* the P38MAPK signaling pathway to achieve immune escape and promote the development of GC (Wang et al., 2021a), which reflects the opposite physiological functions of M1 and M2 *Mφs via* different pathways acting on the same target. MiRNA-223, which can inhibit the proliferation of HCC cells, has been described above. Interestingly, in GC, M-sEVs target PTEN and activate the PI3K/AKT signaling pathway by delivering miRNA-223 and miRNA-21 to GC cells, inhibiting apoptosis, and promoting metastasis of GC cells. MiRNA-223 can also change the actin cytoskeleton and upregulate various proteins related to epithelial-mesenchymal transition (EMT). MiRNA-21 also makes GC resistant to cisplatin (Zheng et al., 2017; Zheng et al., 2020). The role of miRNA-223 in GC is different from that in HCC. One possible explanation is that miRNA-223 can induce *Mφs* to have an anti-tumor or tumor phenotype under different pathological conditions, and has pleiotropic effects in cancer cells (Liu et al., 2020a). It is also possible that different receptors cause *Mφs* to change into a different phenotype with an opposite function. In addition, studies have shown that miRNA-223 not only affects the metastasis of cancer cells but also promotes adriamycin resistance of GC cells by inhibiting the F-box and WD repeat domain 7 (FBXW7) (Gao et al., 2020). Like other malignant cancers, M2 *Mφs* is increased in GC tissues and promotes cancer development. Additionally, tumor suppressor gene T-lymphocyte intracellular antigen 1 (TIA1) is transferred to GC cells by M2-sEVs and induces downregulation of targets through miRNA-487a, which promotes the progression of GC and induces the proliferation and tumorigenesis of GC cells (Yang et al., 2021a). sEVs do not only act on recipient cells through miRNAs and lncRNAs but also play physiological functions by delivering proteins, such as polymorphic apolipoprotein E (ApoE), which plays a key role in cardiovascular and neurodegenerative diseases (MacRitchie et al., 2012) and inhibits tumor growth (Ha et al., 2009; Pencheva et al., 2012). However, other evidence suggests that it is also necessary for the proliferation of cancer cells (Chen et al., 2005). ApoE carried in M2-sEVs promotes the migration of GC cells by activating the PI3K/Akt signaling pathway to reshape the migration of cytoskeleton support (Zheng et al., 2018). In addition to miRNA-21, miRNA-588 can also increase cisplatin resistance in GC. Cylindromatosis (CYLD) is a representative deubiquitinase that plays an important role in multiple cellular processes in tumorigenesis, such as apoptosis, cell cycle, cell migration and DNA damage (Sun, 2010). M2-sEVs lead to cisplatin resistance in GC cells by delivering miRNA-588, which targets CYLD (Cui et al., 2021). Resistance to apoptosis is thought to be responsible for the development of drug resistance. Therefore, these mirRNAs may lead to drug resistance by inhibiting cancer cell apoptosis. Therefore, whether targeted miRNA inhibition can reduce drug resistance and promote apoptosis to achieve therapeutic effects may be the direction of future research on malignant diseases.

### Female-Related Cancers

Women’s health has received increased attention over the years. Here, we mainly review epithelial ovarian cancer (EOC), endometrial cancer, breast cancer, and endometriosis, four important types of female-related cancers. Among the types of ovarian cancer with low incidence and high mortality, EOC is the most common one (Bray et al., 2018). Cyclin-dependent kinase inhibitor 1B (CDKN1B) (P27), a cell cycle regulator, is an inhibitor of cell cycle progression during G1/S transition. CDKN1B is not considered a typical tumor suppressor because it is very lightly mutated in tumors (Chu et al., 2008). Genomic analysis showed that CDKN1B is associated with tumor progression. Among cell cycle-related miRNAs, miRNA-221-3p with the most specific expression can downregulate CDKN1B. Low expression of CDKN1B is associated with poor prognosis and poor overall survival of patients with EOC. Studies have shown that M2-sEVs transfer miRNA-221-3p to tumor cells to target CDKN1B, promote cell proliferation and G1/S conversion, and promote the progression of EOC (Li and Tang, 2020). However, CDKN1B has a more complex upstream regulation mechanism, and miRNA-221-3p is an important but not the only factor in this mechanism. Another study reported that the transfer of miRNA-29a-3p and miRNA-215p by M2-sEVs induces upregulating T cell (Treg)/T helper cell 17 (Th17) ratios and synergistic inhibition of Signal transducer and activator of transcription 3 (STAT3), resulting in an immunosuppressor microenvironment that promotes EOC progression and metastasis, suggesting that the imbalance in T-cell subsets might be associated with poor outcomes (Zhou et al., 2018). This indicates that M-sEVs can not only act on adjacent cells or cancer cells but also on T lymphocytes to affect the disease. The tumor necrosis factor (TNF)-like weak inducer of apoptosis (TWEAK) is a member of the TNF superfamily. TWEAK is a multifunctional cytokine that mediates the anti-tumor effects of tumor-infiltrating *Mφs* by playing a role in the regulation of immune responses, thereby affecting the biological characteristics of multiple cancer cell types (Chicheportiche et al., 1997; Kaduka et al., 2005; Maecker et al., 2005). TWEAK increases the proportion of *Mφs* and miRNA-7 levels in M-sEVs and receptor EOC cells and contributes to miRNA-7 enrichment in *Mφs*, which reduces the activity of the EGFR/AKT/ERK1/2 pathway and ultimately inhibits metastasis of EOC cells (Hu et al., 2017). This suggests that M-sEVs are altered by external stimuli and TWEAK is a potential therapeutic target. M-sEVs can act on tumor cells and vice versa. Studies have shown that the sEVs secreted by TAMs (TAMs-sEVs) inhibit endothelial cell migration by targeting the miRNA-146b-5P/TNF receptor-associated factor 6 (TRAF6)/nuclear factor-κβ(NF-κβ)/Matrix metalloproteinase 2 (MMP-2) pathway. However, EOC-derived sEVs can transfer lncRNAs to *Mφs*, thereby remotely reversing the effect of TAMs on endothelial cells (ECs) and promoting endothelial cell proliferation and migration to establish angiogenesis and tumor metastasis. This finding suggests that the function of *Mφs* can be altered in the presence of tumor cells (Wu et al., 2017). This may explain the metastasis and recurrence of malignant diseases, and also suggests that sometimes the therapeutic effect cannot be achieved *via* M-sEVs alone because of the “counterattack” of tumor cells. It was shown in a previous study that miRNA-223 has different effects in different pathological environments and different recipient cells, which can inhibit the proliferation of HCC cells, promote the metastasis of GC cells, and promote the resistance of GC cells to doxorubicin. In EOC, M-sEVs can transfer miRNA-223 to cancer cells to promote drug resistance by targeting the PTEN/PI3K/AKT pathway (Zhu et al., 2019a), while in breast carcinoma (BCC), miRNA-223 promotes the invasion of BCC cells through the Mef2c-β-catenin pathway (Yang et al., 2011). BCC is the most common malignant tumor in women. The main cause of death is distal metastasis and the incidence of this disease has increased in young women. The increased miRNA-503-3p load in M-sEVs promotes glycolysis and reduces mitochondrial oxidative phosphorylation (OXPHOS) in BCC cells, and activates the Wnt/β-catenin signaling pathway by reducing disheveled-associated binding antagonist of beta-catenin 2 (DACT2) expression, repressing oxygen consumption rate and adenosine-triphosphate (ATP) levels, promoting the malignant phenotype of BCC cells and promoting tumor progression (Huang et al., 2021a). Chemotherapy, one of the important treatment methods for cancer, inhibits tumor development through apoptosis of cancer cells, but this method may change the physiological state of *Mφs* and M-sEVs (King et al., 2012). Researchers have examined the role of M-sEVs in metastasis after chemotherapy and established the post-chemotherapy cancer microenvironment model. M-sEVs that were screened had increased amounts of IL-6 that increases the phosphorylation of STAT3, which likely explains the increased transcription of STAT3 target genes such as CyclinD1, MMP2 and MMP9. The results showed that M-sEVs may promote BCC proliferation and metastasis by activating the IL-6/STAT3 signaling pathway after chemotherapy (Yu et al., 2019), which may be one of the reasons for relapse and metastasis after chemotherapy. These results provide new targets for treatment of malignant tumors after chemotherapy. Long-term endometrial exposure to estrogen is a major risk factor for endometrial cancer. Additionally, diabetes, high blood pressure, age, and obesity are closely related to the occurrence and development of endometrial cancer (Braun et al., 2016). In recent years due to lifestyle changes or estrogen-rich nutrition, there has been an increasing incidence of endometrial cancer. Surgery is the main treatment method, but the cost is high. Nowadays, researchers are committed to revealing the micro pathogenesis of the disease, and studying whether it can provide a new target for endometrial cancer treatment. Underexpression of miRNA-192-5p in M2-sEVs significantly restricts tumor formation, while the expression of its downstream target, the catalytically active protein kinase interleukin 1 receptor (IL-1R)-associated kinase 1 (IRAK1), is positively correlated with cancer progression. The reduction in miRNA-192-5p expression promotes IRAK1 expression in endometrial cancer cells and activates NF-κβ signaling, inhibiting apoptosis and promoting tumor development (Wang et al., 2022b). M2-sEVs can also lead to tumor recurrence by reducing the radiosensitivity of endometrial cancer cells. Not only miRNAs and lnRNAs but also circular RNAs (circRNAs) can affect the disease. It was speculated that hsa_circ_0001610 increases the expression of cyclins, a key regulator of the cell cycle, by acting as a ceRNA of a competitive endogenous target of miRNA-139-5p. Cyclin B1, a key mitotic cyclin, is a downstream target of hsa_circ_0001610/miRNA-139-5p axis. After the transfer of hsa_circ_0001610 by M2-sEVs to endometrial cancer cells, cyclin B1 expression is upregulated through miRNA-139-5p, which affects the G2/M phase of the cell cycle, when the cells are the most sensitive to radiation, reduces the radiosensitivity of endometrial cancer cells, and affects prognosis (Gu et al., 2021) (see Table 2). This will provide new targets for enhancing the radiosensitivity of EC cells in clinical settings. Some diseases are regulated by sEVs from other sources due to the specific location of growth, such as endometriosis (EM), where endometrioid tissue persists and grows outside the uterine cavity (especially in the pelvic cavity), which is a common benign tumor in women. Peritoneal *Mφs* (pM-sEVs) are closely related to EM (Capobianco and Rovere-Querini, 2013). PM-sEVs regulate silencing information regulator 2 related enzyme 1 (SIRT1)/NF-κβ and miRNA-610/MDM2 signaling pathways through miRNA-22-3p and lncRNA CHL1-AS1, respectively, to enhance the proliferation, migration, and invasion of ectopic endometrial stromal cells (Zhang et al., 2020; Liu et al., 2021b).

### Pancreas-Related Cancers

Pancreatic cancer (PC) is considered the fourth leading cause of death and is often detected in advanced stages; the most common type being pancreatic ductal adenocarcinoma (PDAC). MiRNA-21-5p is not only an oncogenic miRNA but can also be used to identify pancreatic tumors, as its overexpression is correlated with the overall survival and prognosis of patients with PC (Karasek et al., 2018; Gilles et al., 2019). M2-sEVs directly target Krüppel-like factor 3 (KLF3) *via* the upregulation of miRNA-21-5p to promote PC stem cell differentiation and activity. Upregulation of another oncogenic miRNA, miRNA-501-3p, can lead to the poor expression of TGF-β Receptor III (TGFBR3) *via* the transforming growth factor-β (TGF-β) signaling pathway activation, PC stem cell regulation, and the enhancement of proliferation, migration, invasion, anti-apoptotic ability (Yin et al., 2019; Chang et al., 2021). In brief, miRNA-21-5p and miRNA-501-3p both promote PC cell invasion, migration, metastasis, and tumor formation. X-linked inhibitor of apoptosis protein (XIAP) silencing can increase the apoptosis of PC cells stimulated by TNF-associated apoptosis-inducing ligands. The recently discovered lncRNA set-binding factor 2 antisense RNA 1 (lncRNA SBF2-AS1) is found in high levels in M2-sEVs and as a competitive endogenous RNA, it can inhibit the expression of miRNA-122-5p and increase the expression of XIAP, promoting the tumorigenic ability of PC cells (Yin et al., 2020). Contrarily, lncRNA SBF2-AS1 silencing can restrain the tumorigenic ability of PC cells. Gemcitabine analog is the first-line drug for PDAC, inhibiting cell growth by inhibiting DNA replication. In addition to lncRNA SBF2-AS1, gemcitabine resistance can also be induced by the transfer of miRNA-365 to PDAC cells to upregulate triphosphate-nucleotide (NTP). Increased levels of NTP upregulates cytidine deaminase (CDA), an enzyme responsible for gemcitabine inactivation in humans (Binenbaum et al., 2018). However, at present there are few studies on gemcitabine resistance caused by lncRNA SBF2-AS1 delivered by M2-sEVs. Dysregulation of E2F expression has been demonstrated in many cancers, including PDAC. Among the early 2 factor (E2F) family, early 2 factor 2 (E2F2) expression is negatively correlated with angiogenesis, and the overexpression of miRNA-155-5p and miRNA-221 can promote angiogenesis and lead to tumor development. Studies have shown that M2 *Mφs* can not only increase the expression of miRNA-155-5p and miRNA-221-5p in sEVs but also inhibit the expression of E2F2 in ECs, thereby promoting angiogenesis, leading to the progression of PDAC (Yang et al., 2021b). This may explain the poor efficacy of traditional anti-VEGF drugs in PC and provide hope for the application of anti-angiogenesis therapy with PDAC.

### Other Tumors

M-sEVs affect disease development or drug resistance in various other tumors in different ways. For example, M2-sEVs enable complementary pairing of miRNA-31-5p and large tumor suppressor kinase 2 (LATS2)-encoding sequences in recipient oral squamous cell carcinoma (OSCC) cells. Thus, LATS2 expression is inhibited and Hippo signaling is inactivated to support OSCC growth (Yuan et al., 2021). Interestingly, M-sEVs can interact closely and be taken up by OSCC cells within a very short time and their uptake is time-dependent. SEVs derived from THP-1 (THP-sEVs) and primary human *Mφs* (PHM-sEVs) can activate the AKT/glycogen synthase kinase-3β (GSK-3β) signaling pathway through the promotion of proliferation, regulation of cell cycle and suppression of apoptosis and reduces the sensitivity of OSCC cells to chemotherapeutic drugs (Tomita et al., 2020). While THP-sEVs contain higher levels of chemokines than PHM-sEVs, these chemokines may promote the migration and invasion of cancer cells. Therefore, THP-sEVs promote the migration and invasion of cancer cells, but similar effects were not observed after cancer cells were exposed to PHM-sEVs. This shows that compared with PHM-sEVs, THP-sEVs are more likely to promote cancer cell malignancy. Therefore, the functional contents of M-sEVs need to be further studied. In colon cancer (CC), M2-sEVs facilitate CC cell proliferation, invasion, and metastasis through miRNA-183-5p by targeting THEM4-mediated PI3K/AKT and NF-κβ pathways, and inhibit T cell immune response, promote cell migration, and invasion, as well as immune escape, through miRNA-155-5p by downregulating ZC3H12B-mediated upregulation of IL-6 (Zhang et al., 2021b; Ma et al., 2021). In addition to the tumor cells, ECs, and T cells mentioned above, and M-sEVs can also be transported to smooth muscle cells (SMs). Studies have shown that miRNA-155-5p from TAMs-sEVs promotes the formation of unruptured intracranial aneurysms (IAs) and TAM infiltration through the targeted inhibition of Gremlin 1 (a secretory bone morphogenetic protein antagonist) in SMs (Feng et al., 2019). This indicates that the M-sEVs do not only influence the development of diseases with a similar origin, such as tumors, but also affect the pathogenesis of unrelated diseases (see Table 1). Therefore, whether clinical sEV inhibitors can be used to treat certain diseases with or without side effects, warrants further research. Actin filament associated protein 1 antisense RNA1 (LncRNA AFAP1-AS1) delivered by M2-sEVs can downregulate miRNA-26a and upregulate activating transcription factor 2 (ATF2) to affect the migration and metastasis of esophageal cancer cells, as well as *in vitro* lung tumor metastasis in EC (Mi et al., 2020). In addition to the most studied miRNAs and lncRNAs, proteins secreted by M-sEVs are also involved in tumor development. Widely expressed membrane proteins, like disintegrin and metalloproteinase 15 (ADAM15), are released into the extracellular space to directly inhibit tumor development without triggering other immune cells (Lee et al., 2012) (see Table 2). This suggests that M-sEVs can transmit messages precisely to recipient cells without interfering with other cells. It also provides the basis for the bioengineering of M-sEVs.

## Cardiovascular Diseases

Despite the widespread use of lipid level-lowering drugs and a healthy diet, cardiovascular events caused by the factors, such as high blood pressure, can lead to atherosclerosis (AS) and even myocardial infarction (MI), which are life-threatening in severe cases and are still frequent worldwide. Studies have shown that hypertension can increase the number of *Mφs* in the heart tissue, leading to *Mφs* infiltration into blood vessels. Intercellular cell adhesion molecule-1 (ICAM1) is an adhesive protein expressed by ECs, which is a key factor in vascular inflammation. In experiments on rats, reduced levels of miRNA-17-3p (a negative regulator of ICAM1 expression) in M-sEVs induced by angiotensin II (Ang II) significantly increased the expression of ICAM1 and the pro-inflammatory factor plasminogen activator inhibitor-1 (PAI-1). Interestingly, the same phenomenon is observed in circulating sEVs, where intracellular ICAM1 and PAI-1 expressions are increased, serum sEV proportions are increased, and miRNA17 content of sEVs is decreased in the heart of hypertensive rats. These results suggest that the pro-inflammatory signaling pathway in ECs under hypertension induced in rats by continuous infusion of Ang II is at least partially activated by M-sEVs (Osada-Oka et al., 2017). There are three key bridging molecules for the progression of AS as follows: miRNA-99a, which inhibits the proliferation of hematopoietic stem cells; miRNA-146b, which inhibits the pro-inflammatory activity of M1 *Mφs*; miRNA-378A-3p, which controls the proliferation of *Mφs* under IL-4 stimulation. It was found that the increase in the levels of these molecules in the M-sEV culture with IL-4 significantly increases the function of anti-inflammatory factors like TNF-α and NF-κB. Each of the three miRNAs carried by bone marrow-derived *Mφs* polarized with IL-4 (BMDM-IL-4-sEVs) participates in the regulation of inflammatory responses by targeting different parts of the genome that controls the TNF-α/NF-κB signaling pathway, targeting hematopoietic stem cells (HSCs) and myeloid cells, and enhancing anti-inflammatory capacity in atherosclerotic lesions. In addition to slowing disease progression, BMDM-IL-4-sEVs can also control hyperlipidemia-driven hematopoiesis by limiting the amplification of multipotent progenitors (MPPs), suggesting that they regulate HSC self-renewal and/or differentiation. In general, M-sEVs regulate hematopoiesis and inflammation through miRNA cargo to control the development of AS (Bouchareychas et al., 2020). This suggests that multiple miRNAs derived from the same *Mφs* can act on multiple cells to perform the same physiological functions. In this case, promoting or inhibiting sEVs secretion can greatly improve clinical efficacy. Interestingly, M-sEVs stimulation by not only IL-4 but also by oxidized low-density lipoproteins, high glucose, and nicotine promotes the development of AS through different pathways, which is consistent with a significantly increased risk of AS in smoking patients with an unbalanced diet and diabetes (Bouchareychas et al., 2021; Zhang et al., 2019; Zhu et al., 2019b). In addition, oxidized LDL can also induce M-sEV-mediated miRNA-106a-3p transfer to vascular smooth muscle cells (VSMCs), which directly binds to CASP9, inhibits the caspase signaling pathway in VSMCs, promotes proliferation of human VSMCs, and inhibits apoptosis (Liu et al., 2020c). In untreated or uncontrolled coronary AS, the plaque becomes unstable, detaches, and forms a clot that completely blocks the blood vessel, leading to ischemic necrosis, or acute myocardial infarction (AMI) in the heart muscle fed by the vessel. The family of tissue inhibitors of metalloproteinases (TIMP1˗4) has four members, among which TIMP3, which is downregulated in various cardiovascular diseases, is believed to be beneficial for myocardial overexpression after MI (Fan and Kassiri, 2020; Takawale et al., 2017). Increased miRNA-21-5p content in M1-sEVs in the mouse model of MI promotes myocardial fibrosis and ventricular remodeling by targeting TIMP3 and accelerates apoptosis of myocardial cells (Dong et al., 2021). In addition, miRNA-155 in M1-sEVs is absorbed by cardiac fibroblasts, which inhibits fibroblast proliferation by downregulating the Son of sevenless gene (*Sos1*), which promotes cell proliferation, and increases fibroblast inflammation by decreasing the expression of the anti-inflammatory gene Suppressor of Cytokine Signaling 1 (*Socs1*) (Wang et al., 2017). Interestingly, miRNA-155 in M1-sEVs can be transported not only to fibroblasts, but also to ECs, and inhibit VEGFR 2 signaling and integrin β1 expression by downregulating RAC1/PAK_1/2_ and Sir T1/AMPK_2α-e_NOS pathways (Liu et al., 2020d). MiRNA-155 not only plays a role in MI but also has an impact on uremic cardiomyopathy (UCM) by downregulating FoxO3a, leading to cardiomyocyte (CM) apoptosis, cardiac hypertrophy, and fibrosis in uremic conditions (Wang et al., 2020b). This suggests that the same sEV contents can be transported to different cells, having not only opposite roles (miRNA223 in tumor cells, see Table 1) but also synergistic ones. In the MI microenvironment, M-sEVs can not only affect myocardial cells but also bone marrow mesenchymal stem cells (BMSCs) to promote the development of disease. Studies have shown that M1-sEVs induced by hypoxia/serum deprivation (H/SD) can inhibit the expression of the anti-apoptotic protein B-cell lymphoma (Bcl)-2 by transferring miRNA-222 into BMSCs. Inhibition of BMSC proliferation and migration leads to BMSC apoptosis, which may affect the efficacy of BMSCs in the treatment of AMI. Moreover, these sEVs induce *Mφs* polarization toward the M1 phenotype and form a vicious circle aggravating the disease (Qi et al., 2021). Reperfusion therapy after MI can relieve pain actively and effectively or reduce the extent of infarction and improve prognosis. Ischemia-reperfusion injury often causes cardiac insufficiency and even malignant arrhythmia. In some studies, *in vitro* hypoxic-reoxygenation was used to simulate ischemia-reperfusion injury *in vivo* to study its pathogenesis and provide new therapeutic options. Hypoxia and reoxygenation-induced and LPS-induced *Mφ* polarization to the M1 phenotype lead to the upregulation of miRNA-29a in sEVs and targeted inhibition of myeloid cell leukemia-1 (MCL-1) expression, an anti-apoptotic member, promoting CM pyroapoptosis (Wang et al., 2021b). Later, during the repair period after MI, *Mφs* mainly change from M1 to M2 phenotype, produce anti-inflammatory/repair cytokines, eliminate inflammation, perform appropriate myocardial repair, inhibit abnormal remodeling, and prevent myocardial ischemia/reperfusion injury. MiRNA-148a carried by M2-sEVs, for example, not only alleviates Ca^2+^ overload and dysregulation of some myocardial ischemia markers by lowering proteins closely related to Ca^2+^ development but also inhibits thioredoxin-interacting protein (TXNIP), Toll-like receptor 4 (TLR4)/NF-κβ/NACHT, LRR, and PYD domain-containing protein 3 (NLRP3) inflammasome signaling pathways to reduce myocardial ischemia-reperfusion injury (Dai et al., 2020). A mouse model of AMI was established *via* the ligation of the left anterior descending of the coronary artery (LAD). After the hypoxia model was established, it was found that the expression of the apoptotic factor Bax and caspase-3, which is a common downstream effector of the apoptotic pathway, increased, and that of the apoptotic inhibitor Bcl-2 decreased. M2-sEVs reversed the effects of hypoxia. Subsequently, M2-sEVs carry miRNA-1271-5p, which reduces cardiac apoptosis and promotes cardiac repair by targeting Sex-determining region Y box 6 (*Sox6*) mRNA and downregulating it (Long et al., 2021). Coronary artery stenting is a commonly used intervention after AMI, but due to uncontrolled proliferation of VSMCs, stent implantation often causes vascular injury, which leads to restenosis. M2-sEVs upregulate C-KIT expression in VSMCs by activating the c-Jun/activating protein 1 (AP-1) signaling pathway, increasing the number of VSMCs with the c-KIT^+^ stem cell phenotype, and promoting c-KIT expression and softening of nearby VSMCs. Thus, vascular tissue repair is accelerated, which has a profound impact on subsequent treatment strategies using coronary artery stent technology (Yan et al., 2020). However, the component of M2 *Mφs* responsible for this effect needs to be further studied. During the development of cardiovascular diseases, changes in the microenvironment lead to changes in the polarization of *Mφs*, and sEVs transmit different information to various cells, ultimately affecting the prognosis of the disease.

## Inflammatory Diseases

When inflammatory diseases occur in the body due to various reasons, M-sEVs can mediate the communication between local and systemic cells and regulate the molecular events in recipient cells spatially and temporally, including the regression of inflammation and the reduction of heat hypersensitivity caused by inflammation (McDonald et al., 2014). In the peritoneum of mice with dextran sulfate sodium (DSS)-induced ulcerative colitis (UC), the expression of miRNA-21a-5p secreted by M1-sEVs was increased, which targeted the expression of the cell adhesion molecule E-cadherin and reduced its binding to killer-cell lectin-like receptor G1 (KLRG1), indirectly promoting the activation of group 2 innate lymphoid cells (ILC2s), which are important components of natural immunity, leading to excessive pathogenic Th2 immunoreaction commonly observed in UC (Lu et al., 2021). In the same model, M2b-sEV treatment improve colonic length in mice with colitis, which was more effective than M2a-sEVs and M2c-sEVs, and played a protective role by mediating the increase in Treg cell population and upregulation of IL-4 through the CC chemokine 1 (CCL1)/CC chemokine receptor 8 (CCR8) axis and reducing the production of pro-inflammatory cytokines (IL-1β, IL-6, and IL17a) (Yang et al., 2019). Because of different activating stimuli, different subtypes of macrophages secrete different anti-inflammatory factors or reduce the release of different inflammatory factors, which affect the contents of sEVs and thus have different effects on the development of disease. There are few studies on the functional differences between *Mφs* and their sEVs in different subgroups, and their effects on other diseases need to be further studied. An immune-mediated disease of the peripheral nervous system Guillain-Barre syndrome, the main subtype of which is acute inflammatory demyelinating polyneuropathy, is widely used in animal models of experimental autoimmune neuritis (EAN) to simulate this disease. M1-sEVs are capable of aggravating EAN by directly regulating T cells, promoting Th1 cell differentiation (increased proportion) and effector function (increased IFN-γ expression), and increasing IFN-γ expression by CD8^+^T cells, showing the intriguing idea that sEVs serve as a bridge between innate and adaptive immunity. However, M2-sEVs do not inhibit Th1 response, but still, show the potential to attenuate EAN (Du et al., 2020). This proves that the opposite effects of M1-sEVs and M2-sEVs do not all occur through the same target, which also brings difficulties for future research. Abdominal aortic aneurysm (AAA) is a chronic inflammatory disease with unknown etiology, and there is no effective treatment at present. Perhaps the discovery of sEVs may provide a new direction for the treatment of AAA. Most M-sEVs promote the expression of MMP2 in VSMCs through JNK and P38 pathways, impinging the integrity of the aortic wall, while a small number of sEVs exist in T cells, ECs, and fibroblasts. An sEV inhibitor prevents calcium phosphate (CaPO_4_)-induced AAA development, which may be a potential drug for the treatment of AAA (Wang et al., 2019c). As a common clinical lung disease, acute lung injury (ALI) may develop into acute respiratory distress syndrome due to pulmonary fibrosis repair and lead to death. The severity and outcome of the disease are determined by the timing and degree of M2 *Mφs* polarization. After ALI was induced with lipopolysaccharide (LPS) in mice, sEV populations in bronchoalveolar lavage fluid (BALF) were analyzed at different time points following treatment. *Mφs* are the major cells to secrete pro-inflammatory cytokines. M-sEVs can produce a variety of pro-inflammatory cytokines and activate neutrophils to secrete IL-10 in BALF-sEVs at an early stage. In turn, IL-10 polarizes *Mφs* into M2c, leading to fibrosis after ALI (Ye et al., 2020). The bidirectional effect of immune cells and M-sEVs is often important for the prognosis of diseases. It is speculated that blocking neutrophil-derived IL-10 could suppress M2c polarization of *Mφs* and thus reduce fibrosis post ALI. In another study, the body’s response inhibits the development of the disease from another aspect; IL-25 secreted by lung epithelial cells can downregulate the expression of Rab27a and Rab27b in *Mφs*, leading to the inhibition of M-sEV release. Thus, the expression and secretion of TNF-α induced by sEVs are weakened, and further development of the disease is slowed down (Li et al., 2018). Si0_2_ can induce a significant increase in M-sEV secretion in silicosis models of pulmonary fibrosis and inflammation induced by Si0_2_ in mice. Si0_2_-sEVs can effectively promote the development of myofibroblasts, and inhibition of endoplasmic reticulum stress can reverse the fibrotic phenotype of activated myofibroblasts. The inhibitor of sEVs can inhibit Si0_2_-induced pulmonary fibrosis and inflammation, suggesting that M-sEVs may be a therapeutic target for silicosis (Qin et al., 2021). Although it was not proved what components in sEVs caused pulmonary fibrosis, in another study, it was shown that proteins contained in sEVs may affect pulmonary fibrosis. For example, in an *in vitro* model of silicosis established, it was demonstrated that fibroblasts can be induced to transdifferentiate into myofibroblasts by M-sEVs. After secreted phosphoprotein 1 (SPP1) transfer, a marker of pulmonary fibrosis, to fibroblasts *via* M-sEVs, it activates the downstream cascade and leads to myofibroblast transition (FMT), promoting disease development, but the downstream reaction of SPP1 needs to be further studied (Huang et al., 2021b). The researchers established a rat model of pulmonary interstitial fibrosis *via* intratracheal perfusion of bleomycin. Family with sequence similarity 13, member A (FAM13A) is believed to be involved in lung function, and silencing FAM13A enhances the proliferation of pulmonary interstitial fibroblasts. After being absorbed by lung fibroblasts, miRNA-328 in M2-sEVs upregulate the expression of collagen 1A, collagen 3A, and α-smooth muscle actin (α-SMA) by downregulating the expression of FAM13A, thereby promoting the progression of pulmonary fibrosis (Yao et al., 2019). A growing number of studies suggest that elevated Ang II/angiotensin II Type 1 receptor (AT1R) levels exacerbate pulmonary fibrosis. In rat models generated the same way, Ang II stimulated *Mφs* to release AT1R-rich sEVs, and by directly transferring AT1R to fibroblasts, upregulated the TGF-β/Small pathway against the decapentaplegic 2 (Smad2)/Smad3 pathway, which is closely related to the fibrosis of various organs, to promote collagen synthesis, fibroblast activation, and pulmonary fibrosis (Sun et al., 2021). There are not only pro-fibrotic M-sEVs but also anti-fibrotic ones. Studies have shown that the anti-fibrosis properties of M-sEVs are partly due to the overexpression of miRNA-142-3p in alveolar epithelial cells and lung fibroblasts, which reduces the expression of transforming growth factor β receptor 1 (TGFβ-R1) and pro-fibrosis genes, and targets excessive deposition of extracellular matrix to slow the progression of pulmonary fibrosis (Guiot et al., 2020). Except for silicosis, asthma is also closely associated with M-sEVs. EMT often occurs in airway remodeling and leads to structural and functional changes in the airway. After establishing an asthma model with ovalbumin, it was demonstrated that miRNA-21-5p expression was upregulated in alveolar M-sEVs, transferred to rat trachea epithelial cells to target Smad7, and affected the pro-fibrotic TGFβ1/Smad signaling pathway, promoting EMT and airway remodeling and exacerbating asthma progression (Li et al., 2021c). MiRNA inhibitors are considered possible therapies for asthma, while some miRNA inducers may be used in the treatment. For example, in asthma animal models established the same way and in platelet-derived growth factor (PDGF-BB)-treated primary mouse airway smooth muscle cells (ASMCs), overexpression of fibroblast growth factor 1 (FGF1) reduces the function of M2-sEVs and miR-370 was poorly expressed in both models but then recovered after M2Φ-Exos treatment, in turn, M2-sEVs can reduce lung injury and inflammation, inhibit ASMC proliferation and airway remodeling, and slow down asthma development by carrying miRNA-370, downregulating FGF1 expression and inhibiting the MAPK/STAT1 axis (Li et al., 2021d). MiRNA-370 inducer may offer a novel insight into asthma treatment. M-sEVs infected with different pathogens, such as *Mycobacterium tuberculosis*, *Mycobacterium bovis*, *Salmonella typhimurium* or *Toxoplasma gondii*, Shiga toxin 2A, *Mycobacterium avium*, *Escherichia coli*, and *Helicobacter pylori*, can stimulate pro-inflammatory responses *in vivo* through different pathways.

Other studies have shown that M-sEVs also play a role in pregnancy. The placenta can produce a pro-inflammatory response to M-sEVs but not to monocytes (Rice et al., 2018). The placenta can internalize M-sEVs and induce pro-inflammatory cytokine production *via* non-contact dependency, then adjust the production of the placenta factor potentially generating a reaction to maternal inflammation and infection, mediating a protective immune response during pregnancy to prevent damage to the fetus. This may be a way of communication between the mother and the placenta (Holder et al., 2016). By overexpressing miRNA-153-3p in deciduae M-sEVs, the indoleamine 2,3-dioxygenase (IDO)/STAT3 pathway can be inhibited to inhibit the proliferation and migration of trophoblastic cells and regulate the behavior of trophoblastic cells (Ying et al., 2020). MiRNA from M-sEVs may keep a balance between the proper function of trophoblasts and the maternal-fetal immune interface, paving a new approach for the development of novel treatments for unexplained recurrent spontaneous abortion (URSA). Studies have shown that M-sEVs can cross not only the placental barrier but also the blood-brain barrier (BBB). Due to the overexpression of intercellular adhesion molecule 1 (ICAM-1) receptor on human brain ECs under inflammatory conditions, M-sEVs inherit lymphocyte function-associated antigen 1 (LFA-1) from their parent cells and interact with ICAM-1, which mediates the lateral migration of M-sEVs crossing the BBB and significantly increases sEVs uptake by ECs. Studies have shown that intravenous administration of M-sEVs can penetrate the BBB and enter the inflamed mouse brain at a higher rate than the healthy brain, which could greatly improve drug delivery to the brain for future treatments (Yuan et al., 2017). However, there is no conclusion regarding their ability to penetrate the blood-choroid plexus or ependymal barrier. All these results lay a foundation for the clinical application of M-sEVs.

## Orthopedic Diseases

M-sEVs play an important role in fracture, muscle injury, osteoarthritis, tendon injury, osteoporosis and other diseases, In fractures, M1 and M2 *Mφs* have the same role in different stages, unlike other diseases. M1 *Mφs* promote osteogenesis in MSCs in the initial pro-inflammatory phase of the disease, while M2 *Mφs* promote osteogenesis in the later stage of the disease. Bone fracture is a common clinical challenge, and osteoblast differentiation is an important process during fracture healing; therefore, MSCs that can differentiate into osteoblasts play an important role. Studies have shown that M1-sEVs overexpressing miRNA-21a-5p can promote the osteogenic differentiation of MSCs at the early stage of pro-inflammatory response. However, the underlying mechanism warrants further research (Liu et al., 2022). During the anti-inflammatory response in the late stages of the disease, miRNA-5106, which is highly enriched in M2-sEVs, is transferred to MSCs and downregulates its targets, which can inhibit the expression of salt-induced kinases 2 and 3 (SIK2 and SIK3) in osteogenic differentiation, promote osteoblast differentiation and bone mineral deposition, and accelerate fracture healing (Xiong et al., 2020). This suggests that M1/M2 *Mφs* provide an overall balance at different stages of the disease, controlling the eventual recovery of organs from inflammation or injury. Fracture healing is not only dependent on MSCs ossification but also closely related to angiogenesis. The timing of angiogenesis is important. If angiogenesis does not occur in the early stages of fracture healing, nonunion can occur even in the late stages. The immunosuppressive and anti-inflammatory molecule adenosine receptor A2A (ADA2AR) is thought to be closely involved in angiogenesis. *In vivo*, CGS21680 (an ADA2AR agonist) not only enhanced the proliferation and migration of vascular endothelial cells (VECs) and promoted angiogenesis during bone healing but also promoted the secretion of M-sEVs. However, ZM241385 (an ADA2AR antagonist) inhibited these functions, thus inhibiting angiogenesis in the early stage of bone healing. It was proved that ADA2AR not only affects VECs but also plays a role in the secretion of M-sEVs during bone healing (Wang et al., 2021c). M-sEVs can not only play an important role in fracture healing but also promote recovery during muscle injury. Abundant miRNA-501 targeting the transcription factor yin yang 1 (YY1) in M2-sEVs promotes the differentiation of myoblasts, improves inflammatory cell infiltration, and contributes to the regeneration of pubococcygeus muscle. M2-sEVs may provide a new potential myotropic mechanism and a potential therapy for promoting the recovery of injured muscles (Zhou et al., 2021). The main characteristics of knee osteoarthritis (KOA) are inflammation and cartilage degeneration. Therefore, the key to the treatment of KOA is to reduce inflammation, protect cartilage and reduce cartilage apoptosis. PI3K/AKT, as a classical anti-apoptotic pathway, can combat cartilage apoptosis in osteoarthritis by regulating multiple downstream pathways. Studies have confirmed that M2-sEV intervention reduces the expression of PI3K/Akt/mTOR in KOA model rats (Da-Wa et al., 2021). After tendon injury (TI), the natural tendon structure cannot be reestablished, and fibrosis limits normal physiological function. High expression of cirRNA-EP400 in M2-sEVs promotes FGF1/7/9 expression in fibroblasts and tendinocytes by inhibiting miRNA-15b-5p. The expressions of COL-I, COL-III, α-SMA, and TGF-β1 in tendinocytes and fibroblasts increase to promote fibrosis, proliferation, and migration of fibroblasts and tendinocytes after TI and ultimately promote perineural fibrosis after tendon injury (Da-Wa et al., 2021). In addition to this pathway, M-sEVs can also directly target the expression of Smad7 by secreting miRNA-21-5p, activating the TGF-β1 pathway in the tendon, and inducing perineural fibrosis after TI (Cui et al., 2019). After establishing a mouse model of postmenopausal osteoporosis (OP) using oophorectomy (OVX), the mice presented with reduced bone mineral density and reduced bone trabecular number. After treatment with M1-sEVs, bone loss was aggravated. Later experiments proved that M1-sEVs downregulated the dual-specificity protein phosphatase-1 (DUSP1) through the high expression of miRNA-98, which not only inhibited the osteogenic differentiation of osteoblasts but also activated the JNK signaling pathway to aggravate bone loss and OP (Yu et al., 2021).

## Metabolic Diseases

Type 2 diabetes mellitus (T2DM), a common metabolic disease, is characterized by insulin resistance and lipid metabolism disorders. The pathogenic factors are often related to lifestyle habits. Without intervention, serious complications often occur. NADH dehydrogenase (ubiquinone) 1 alpha subcomplex 4 (NDUFA4) mediates the uptake of glucose by adipocytes and the activity of the mitochondrial continuous intravenous infusions (CIV). Under high glucose conditions, miRNA-210 contained in sEVs derived from adipose tissue *Mφs* (ATMs-sEVs) directly binds to *NDUFA4* and inhibits its expression in adipocytes, impinges glucose uptake and mitochondrial CIV complex activity of adipocytes, promotes insulin resistance and obesity, and promotes diabetic obesity pathogenesis (Tian et al., 2020). ATMs-sEVs in obese (OATMs-sEVs) or non-lean individuals contain miRNA-29a, which directly targets peroxisome proliferator-activated receptors (PPAR-δ) by transferring it to fat, muscle, and liver cells, impelling glucose uptake by fat and muscle cells, promoting glucose output in response to insulin, reducing cellular and systemic insulin sensitivity, and promoting obesity-induced insulin resistance (Liu et al., 2019). Obese mice and humans have an increased number of ATMs, which is positively correlated with adiposity. Therefore, whether the intervention of ATMs or ATMs-sEVs can reduce weight or treat diabetes warrants further research, and the study of the key miRNA/miRNAs within lean ATM-Exos (LATMs-sEVs) improving insulin sensitivity is worthy of future investigation. LATM-Exos may become a therapeutic option just as shown in a previous study. When OATM-sEVs and LATMs-sEVs were used to treat lean or obese insulin-sensitive mice, respectively, their insulin levels changed. Insulin-sensitive mice showed insulin resistance and glucose intolerance, whereas insulin-resistant mice showed near normalization of glucose tolerance and improved insulin sensitivity. These results indicate that miRNAs in ATM-sEVs can be taken up by insulin target cells and have a strong effect on cellular insulin function, insulin sensitivity *in vivo*, and overall glucose homeostasis. In addition, miRNA-155 in OATM-sEVs is absorbed by insulin target cells *in vivo* and *in vitro*. It directly inhibits the expression of *PPAR-γ*, insulin signaling, and glucose tolerance, leading to cellular and systemic insulin resistance and glucose intolerance (Ying et al., 2017). Besides the effects of ATM-sEVs on insulin function, studies have shown that bone marrow-derived M-sEVs (BMM-sEVs) also play a role in the development of diabetes. MiRNA-143-5p contained in BMM-sEVs induced by a high-fat diet directly targets the mitogen-activated protein kinase phosphatase-5 (*Mkp5*) mRNA, a negative regulator of insulin resistance, after being transferred to liver cells, and significantly inhibits its expression, thus regulating the insulin signaling pathway and promoting insulin resistance in liver cells. In addition, these BMMs polarized toward M1 pro-inflammatory *Mφs* were found in previous studies and further led to inflammation. Except for liver cells, BMM-sEVs also affect islet β cells. BMM-sEVs transfer miRNA-212-5p to adjacent β cells dor insulin secretion, downregulating sirtuin 2 (SIRT2) in recipient β cells, impairing the SIRT2-mediated Akt/glycogen synthase kinase-3β (GSK-3β)/β-catenin pathway, finally disrupting insulin secretion and glucose intolerance in islet β cells (Li et al., 2021e; Qian et al., 2021). Like other *Mφs*, BMM1-sEVs enhance insulin resistance; however, BMM2-sEVs enhance insulin sensitivity and reduce glucose intolerance and insulin resistance. *NADK* encodes NAD^+^ kinase, which converts nicotinamide adenine dinucleotide (NAD^+^) to NADP^+^ and inhibits insulin, and the intake of *NADK* promotes an anti-inflammatory state. After being transferred to adipocytes, myocytes, and hepatocytes, miRNA-690 in BMM2-sEVs inhibits *NADK* mRNA and improves insulin sensitivity. In addition, treatment with miRNA-690 reduces obesity-related adipose tissue inflammation and promotes repolarization of *Mφs* from the M1 to M2 phenotype, which also contributes to improved insulin sensitivity. Therefore, the miRNA-690-*NADK* axis can regulate cellular insulin activity and play a role as an insulin sensitizer (Ying et al., 2021). M-sEVs can affect not only the development of diabetes but also its complications. For example, patients with diabetes have a significantly increased risk of nonunion and delayed fracture union compared with those without diabetes. When miRNA-144-5p is overexpressed in BMSCs, the expression levels of a large number of osteogenic markers are significantly reduced, indicating that the miRNA-144-5p can be transferred to BMSCs by sEVs derived from diabetic bone marrow-derived macrophages (dBMDM-sEVd) and prevent fracture healing by reducing the expression of Smad1 involved in osteogenic regulation (Zhang et al., 2021c). However, further studies are needed to prove whether miRNA derived from sEVs can promote fracture healing mentioned above and have an impact on diabetic fractures, such as miRNA-21a-5p and miRNA-5106. Diabetic nephropathy (DN), another complication, is closely related to M-sEVs. *Mφs* treated with high glucose produce more sEVs. When these sEVs were injected into mice, TGF-β1/Smad3 pathway was activated in mesangial cells, α-SMA expression increased, morphology and function of mesangial cells changed, and inflammatory factors accumulated in the kidney. This suggests that *TGFB1* mRNA transfer from M-sEVs to mesangial cells activates the TGF-β1/Smad3 pathway, induces mesangial cell proliferation, and promotes fibrosis, as well as accumulation of inflammatory factors (Zhu et al., 2019c). M-sEVs can not only affect mesangial cells but also glomerular visceral epithelial cells (GVEs) and affect disease development. MiRNA-25-3p contained in M2-sEVs is transferred to GVEs and binds to the negative regulator of apoptosis *DUSP1* to inhibit its expression and activate the autophagy of GVEs, thereby protecting the GVEs from high glucose-induced damage (Huang et al., 2020). In addition to DN, M-sEVs can also recover other complications, such as diabetic wound dysfunction, which takes longer due to long-term inflammation and may require amputation in severe cases. M-sEVs can significantly reduce the secretion of pro-inflammatory cytokines TNF-α and IL-6, promote the proliferation and migration of human umbilical vein ECs (HUVECs), and enhance the reduction of wound size, thereby improving angiogenesis and epithelial regeneration of diabetic wounds with type 1 diabetes. In addition, p-Akt is significantly activated after M-sEVs injection, and MMP-9 expression decreases sharply. This also helps fast wound healing. In summary, M-sEVs promote the success of diabetes treatment and its complications in a variety of ways, which may be a new therapeutic strategy for wounds or injuries due to diabetes (Li et al., 2019). This experiment did not determine which *Mφs* the sEVs came from, but we speculate that it is M2 *Mφs*. M2-sEVs just like the M2 *Mφs*, can inhibit the release of inflammatory cytokines and have an anti-inflammatory effect, but whether they release anti-inflammatory factors need further validation. Additionally, more studies are warranted to reveal the contents of sEVs that have an anti-inflammatory effect. Moreover, whether these sEVs can also promote wound healing in T2DM needs further investigation.

## Other Diseases

The research on M-sEVs is not only limited to the above diseases but also includes many other diseases, such as many viral diseases, including HBV, HCV, HIV, syphilis, and prions. M-sEVs also play an important role in many specific diseases, such as the formation of hypertrophic scars, metabolic dysfunction caused by impaired breathing due to sleeping disorders, hemorrhagic shock, septic shock, slow transit constipation, and mucosal damage. In addition to LPS stimulation, other external stimuli may have different effects on M-sEVs and various target cells, as well as some biological processes. For example, calcium oxalate, copper and cobalt ion, zinc ion, deoxycholic acid, and leishmania protozoa stimulation. These studies prove that the regulation of M-sEVs through artificial intervention can be used as a new method for disease treatment.

## Bioengineering

M-sEVs can enhance the therapeutic effect of drugs and vaccines by changing the microenvironment. They can also be used as natural nanocarriers to deliver drugs through biological engineering, helping drugs pass through natural barriers and greatly reducing adverse reactions in drug treatment. M1-sEVs can induce many naive *Mφs* into the M1 phenotype, and these activated M1 *Mφs* can produce more sEVs to activate more naive *Mφs*. Through this cycle, M1-sEVs play a key role in innate and adaptive immune responses. Studies have shown that M1-sEVs in combination with vaccines can accumulate in the lymph nodes, create a local inflammatory environment suitable for inducing Th1 adaptive immune response, enhance vaccine efficacy in growth inhibition, which can be used as a new type of immune adjuvant for cancer and infectious lymphatic tissues, and exert strong tumor disease vaccines (Cheng et al., 2017). When M1-sEVs were used as the carrier of paclitaxel (PTX) to prepare nanomaterials (PTX-M1-sEVs), it could not only deliver a large amount of PTX to the tumor site safely and effectively but also activate the NF-κβ pathway and enhance the pro-inflammatory environment and anti-tumor activity. PTX-M1-sEVs combined with aminoethyl aminoamido-polyethylene glycol (AA-PEG) in the treatment of lung metastases not only target the σ receptor overexpressed by LC cells and specifically deliver the cytotoxic payload to tumor cells avoiding healthy tissues but also increase circulation time of the drug in the blood. M-sEV film-coated polylactic acid-glycolic acid copolymer nanoparticles immobilize the mesenchymal-epithelial transformation-binding peptide (c-MET), which has a high affinity for c-MET overexpressed in triple-negative breast cancer cells. A new drug delivery system (MEP-D) has been developed to evaluate its biosafety, as well as its accumulation in tumor tissues and anti-tumor efficacy *in vitro* and *in vivo*, with excellent results (Kim et al., 2018; Wang et al., 2019d; Li et al., 2020c). All these could circumvent some limitations such as poor targeting and toxicity of chemotherapeutic, and avoid affecting the efficacy of chemotherapy and surgery. Apart from PTX, researchers are also using M1-sEVs containing gemcitabine and deferasirox in the treatment of PC, ultimately overcoming drug resistance and improving its therapeutic potential to achieve effective delivery of drugs, which is a promising new anti-cancer strategy (Zhao et al., 2021). However, due to the disadvantages of sEV separation technology, such as low yield and loss of function, some studies proposed redesigning immune sEVs with synthetic liposomes and hybridizing M-sEVs with synthetic liposomes to construct vesicles with a size of less than 200 nm, called hybrid sEVs (HEs). The results proved that drug-loaded HEs are more stable and toxic to cancer cells with enhanced pH-sensitive drug release under acidic conditions, facilitating drug delivery to acidic cancer environments, maximizing intracellular drug delivery, and facilitating mass production of M-sEVs (Rayamajhi et al., 2019). Because the large particle size of quantum dots (QDs) commonly used to label luminaries results in significant steric hindrance, DNA hinges (functionalized DNA) have been designed to anchor QDs to the sEVs surface, enabling a mild and biologically compatible labeling method. sEVs-DNA-QDs are rapidly phagocytosed by tumor cells, suggesting that they can be used for tumor labeling. In addition, the internal vesicle space of sEVs can also be used as a drug carrier. Due to the limitation of the low separation rate of sEVs, artificial vesicles of M1 *Mφs* (M1mvs) driven by a pneumatic liposome extrusion system were constructed. The results showed that M1mvs could kill tumor cells. Therefore, is it possible to design specific sEVs-DNA-QDs for tumor markers on M1mvs, which can be used both for treatment and imaging (Fan et al., 2019). M1mv-sEVs-DNA-QDs not only solve the problem of low sEV production but can also establish targeted therapy of tumor cells, greatly reduce the side effects of chemotherapy drugs, and improve the bioavailability of drugs, which suggests that these programmable drug delivery vesicles have broad application prospects in a variety of tumors. In addition to drug vectors, TAMs have been reprogrammed into M1 *Mφs* to promote M1 polarization and target IL4 receptors to inhibit tumor growth by M1-sEVs. This may serve as a new type of cancer immunotherapy (Gunassekaran et al., 2021). The greatly increased M1/M2 *Mφs* ratio inhibits the development of tumors. Similarly, in inflammatory diseases, whether the M2/M1 *Mφ* ratio can be increased to slow down the disease may be a future research direction. In order to eliminate the toxic side effects, such as the rapid elimination of contrast agents (CAs) by the immune system and renal damage excretion, studies have also used M-sEVs as a contrast agent for MRI. Reconstructing EVs with Gadolinium (Gd) to maximize the delivery of Gd to the target site resulted in enhanced contrast in the vascular system and a high retention time to develop biomimetic CAs for enhanced MRI. It has high colloidal stability and retains protein characteristics and molecular functions, showing specificity to cancer cells (Rayamajhi et al., 2020). M-sEV bioengineering has a high clinical value, making use of the special structure and targeting of EVs.

## Compared With sEVs Derived From Dendritic Cells

Dendritic cells, as a major specialized antigen-presenting cell, like macrophages, can also alter their physiological state by transferring sEVs contents or by triggering cellular signals at the cell surface. sEVs derived from dendritic cells (DCs-sEVs) have the same basic structure and the same type of contents as other EVs (including M-sEVs). The difference is that compared to *Mφs*, DCs are weak in phagocytosis, but have more antigen presenting cells. The characteristics of parental cells determine the function of sEVs derived from them, so the main role of DCs-sEVs is to act as small antigen presenting entities that activate immune cells such as T cells and NK cells to amplify the immune response. Specifically, DCs-sEVs are enriched in major histocompatibility complex (MHC) class I and II molecules, which are believed as protein markers in the sEVs released upon maturation, that can respectively stimulate CD8^+^ and CD4^+^ T cells, bridging the innate and adaptive immune responses, indicating that DCs-sEVs, similar to parental cells, can carry intact antigens as well as peptides capable of activating antigen-specific T cells (Lindenbergh and Stoorvogel, 2018). In addition to MHC molecules, DCs-sEVs also carry costimulatory molecules (CD80, CD86, and CD40), integrin αvβ2 (αvβ2), Intercellular adhesion molecule 1 (ICAM-1), and later studies have proposed “true” exosomal protein markers from dendritic cells such as tetraspanins (CD9, CD63, and CD81), bear the proteins syntenin-1 and the tumor susceptibility gene 101 (TSG101) (Segura et al., 2005; Kowal et al., 2016). The activation of T cells by antigen-carrying DC-derived sEVs (DCs-sEVs-An) is dependent on host B cells, and it is hypothesized that these B cells could act as both activators and transporters in this system. In immature DCs (imDCs), ubiquitinated MHC class II molecules are sorted into MVBs for transfer to and degradation in the lysosome, suggesting that sEV-associated MHC-II is associated with plasma membrane-derived microvesicles, but that upon maturation of these cells in the presence of antigen-specific T cells, MHC class II molecules are packaged into ILV and finally released as sEVs (Buschow et al., 2009). Mature DCs (mDCs) and immature DCs secrete mDCs-sEVs and imDCs-EVs, respectively. ImDCs internalize sEVs more efficiently, forming antigen-MHC complexes that are later transported back to the DC surface for presentation to T cells. MDCs may retain more sEVs on their surface, further exogenously activating imDCs and T cells (Montecalvo et al., 2008). Unlike M1-sEVs and M2-sEVs, which have opposite roles in most diseases, mDC-EVs and imDC-EVs act in the same way, both stimulating T cell proliferation and inducing and amplifying antigen-specific responses, T cell memory and antitumor immunity, but mDC-EVs are more intense and stimulating. The T cell activation induced by imDC-EVs leads to different functional outcomes: T cells activated by small and medium EVs tend to have a Th1 phenotype, while large EVs bias T cells towards a Th2 phenotype (Tkach et al., 2017). This feature is not possessed by M-sEVs. The production and release of DCs-sEVs is also dependent on changes with the surrounding environment and the maturation stage of parental cells, including DCs, stimulatory signals generated by T cells and DNA damage processing. For example, mDC-EVs are relatively enriched in CD86 and ICAM-1, whereas imDC-EVs carry more lactolipid globule-epithelial growth factor-factor VIII (MFG-E8) (Segura et al., 2005). There are three pathways by which DCs-sEVs can exert their physiological functions. First, DCs-sEVs can rely on the activation of other immune cells to perform their functions, such as direct antigen presentation to T cells, which is also considered as a restimulation of activated T cells. Secondly, DCs-sEVs can also mediate indirectly antigen presentation to T cells, where DC-EVs with ICAM-1 on their surface can be captured by other DCs through binding to LFA-1. Keeping them on their surface, where they can present peptide-laden MHC complexes directly to CD4^+^ and CD8^+^ T cells. This mechanism would allow amplification of T cell responses by cross-modification of bystander DCs that are not in direct contact with the antigen. Third, DCs-sEVs can be internalized by tumor cells and convert them into stronger immune targets for effector immune cells. Thus DCs-EVs can amplify the mechanism of immune response by transmitting immunomodulatory signals away from their generating sites and do not require parental dendritic cells to maintain stimulation of other immune cells (Pitt et al., 2016). While M-sEVs mostly act directly on tumor cells, or endothelial cells, hepatocytes, etc. to act by directly inhibiting or promoting their differentiation and proliferation. For immune cells, while M-sEVs act on T cells, more often to promote immune escape. DCs-sEVs has immunostimulatory characteristics, and it is not certain whether sEVs derived from different subpopulations of DCs induce different quality of immune responses, but it is certain that, DCs-sEVs has significant advantages over DC-based immunotherapies, and it has been shown that DCs-sEVs containing tumor peptides induce specific anti-tumor immune responses *in vivo*, enhance the ability of specific cytotoxic T lymphocytes and kill tumor cells, showing tumor eradication. It can be applied not only in immunotherapy of tumors, but also to enhance the ability of protective immune response of vaccines, which shows that the modification of DCs-EVs powerfully enhances the immune response, not only affecting immune cells, but also acting on tumor cells (Bu et al., 2015; Lu et al., 2017; Chen et al., 2018). The immunosuppressive phenotype of DCs-sEVs has been shown to reduce systemic inflammation, a role that has been applied to graft repertoire, increasing the survival of allogeneic grafts. DCs-EVs and M-sEVs have similarities and differences in their effects on disease progression, for example, in fracture disease, DC-EVs are internalized by human BMSCs, but have no significant effect on their proliferation or on affecting their osteogenic/chondrogenic differentiation, but can promote cell recruitment and thus stimulate new tissue regeneration. M-sEVs, on the other hand, affect disease by influencing osteogenic/chondrogenic differentiation. In tumor diseases, DCs-EVs powerful and potent ability to amplify immune responses and kill tumor cells is not possessed by M-sEVs. However, M-sEVs act on a wide variety of cells and are not limited to immune cells. In addition, in contrast to dendritic cells, M-sEVs often have a two-sided effect on disease development, with differently polarized M-sEVs having opposite effects in most cases. Both can also end up with the same effect in the same disease through different pathways. These properties lead to the possibility that both may coordinate with each other in the future development, acting together to inhibit disease progression and ultimately to heal the patient.

## Conclusion

**FIGURE 2:**
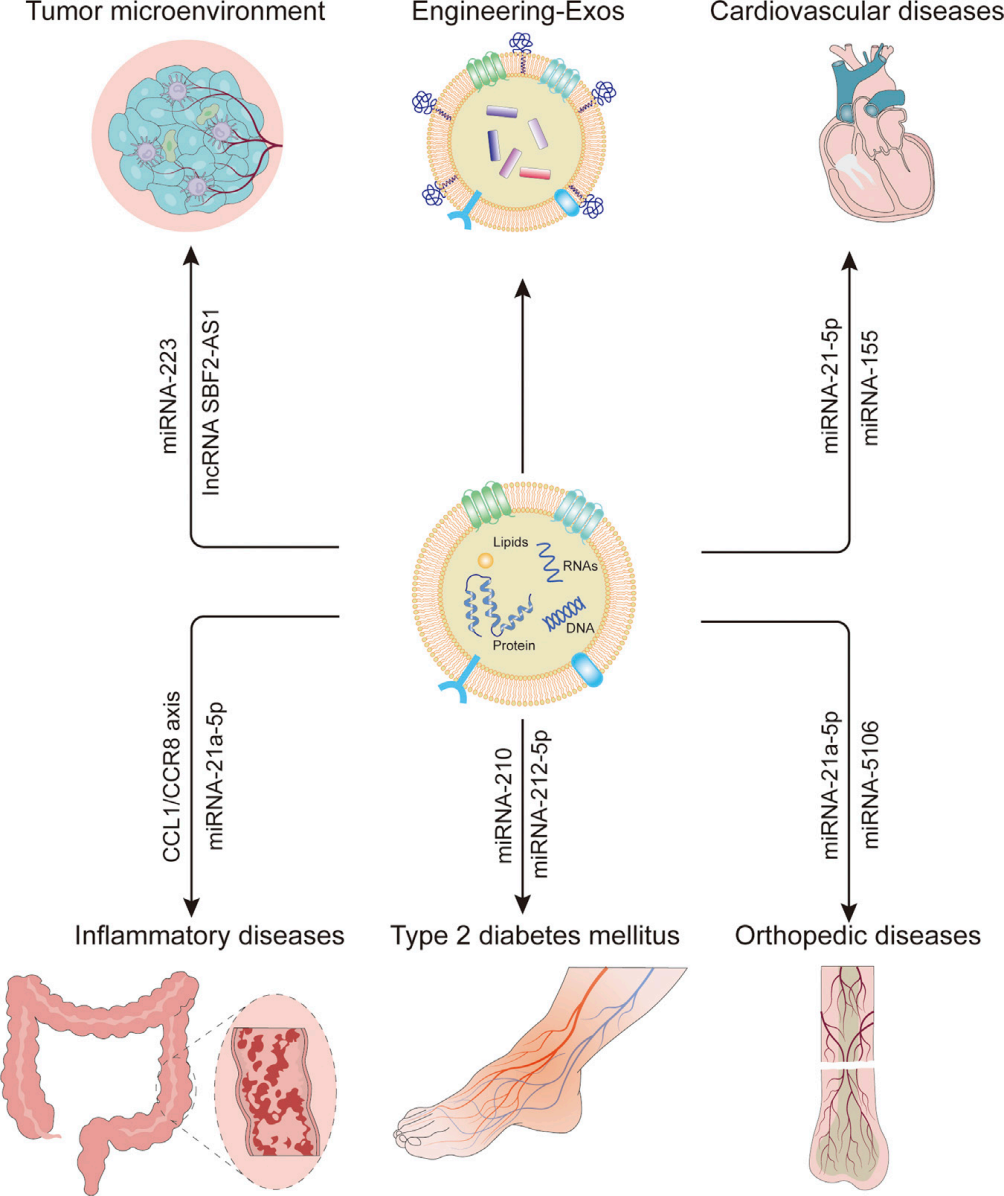
Macrophage-derived exosomes affect disease and applications. M-Exos contain different types of contents that have different functions in multifarious diseases *in vivo*. In addition, they cannot only be used as drug carriers but also as biomarkers and can change the microenvironment to enhance drug efficacy.

M-sEVs play vital roles in the mechanism of the above disease by interfering with tumor growth, angiogenesis, cell proliferation, apoptosis, differentiation, immune response, and inflammatory factors and others. They cannot only be used as drug carriers but can also change the microenvironment to enhance drug efficacy (see Figure 2). However, most studies were conducted in animals or vitro and whether comparable effects will be observed in human studies remains to be assessed. More studies are warranted to prove whether the artificial recombination or reconstruction of sEVs can be safely applied on a large scale given the disadvantages of low yield and functional loss of sEVs. The cellular microenvironment changes dynamically. Different polarized *Mφs* secrete different amounts of sEVs, while sEVs containing different genes act on different receptors and have different physiological functions in different microenvironments which has largely hampered their characterization and manipulation of their properties and functions (see Table 3). Therefore, when interfering with any one of these, it may be difficult to determine which ones to target. Blocking or enhancing one small part of a variety of pathways is a potential treatment option, such as blocking sEV release or changing their contents or the microenvironment, which warrants more research. However, it may not possible to simply intervene on disease-suppressing or disease-promoting sEVs because it may interfere with the normal communication of other contents with the target cells and cause other unexpected complications. At present, a large number of studies only focus on sEVs carrying one or more contents. Whether other contents also play a role is the focus of future research. It is believed that M-sEVs can play an important role in all aspects of medical treatment in the future development of biotechnology.

## Data Availability

No data, models, or code were generated or used during the study.
